# Tetraspanins Function as Regulators of Cellular Signaling

**DOI:** 10.3389/fcell.2017.00034

**Published:** 2017-04-06

**Authors:** Christina M. Termini, Jennifer M. Gillette

**Affiliations:** Department of Pathology, University of New Mexico Health Sciences CenterAlbuquerque, NM, USA

**Keywords:** tetraspanins, signal transduction, tetraspanin-enriched microdomains, adhesion-mediated signaling, receptor-mediated signal transduction

## Abstract

Tetraspanins are molecular scaffolds that distribute proteins into highly organized microdomains consisting of adhesion, signaling, and adaptor proteins. Many reports have identified interactions between tetraspanins and signaling molecules, finding unique downstream cellular consequences. In this review, we will explore these interactions as well as the specific cellular responses to signal activation, focusing on tetraspanin regulation of adhesion-mediated (integrins/FAK), receptor-mediated (EGFR, TNF-α, c-Met, c-Kit), and intracellular signaling (PKC, PI4K, β-catenin). Additionally, we will summarize our current understanding for how tetraspanin post-translational modifications (palmitoylation, N-linked glycosylation, and ubiquitination) can regulate signal propagation. Many of the studies outlined in this review suggest that tetraspanins offer a potential therapeutic target to modulate aberrant signal transduction pathways that directly impact a host of cellular behaviors and disease states.

## Introduction

Tetraspanins are membrane-spanning proteins with a conserved structure that function primarily as membrane protein organizers. Phylogenetic analysis identified 33 tetraspanins in humans, 37 in *Drosophila melanogaster* (Charrin et al., 2014), and 20 in *Caenorhabditis elegans* (Huang et al., 2005), while only 17 were identified in *Arabidopsis thaliana* (Boavida et al., 2013). Tetraspanins have also been identified in the ameoba, *Dictyostelium discoideum*, which exists as both a unicellular and multicellular organism (Albers et al., 2016). While some tetraspanins are expressed ubiquitously in humans, others are cell or tissue specific (Maecker et al., 1997; de Winde et al., 2015), providing a means to regulate the signal transduction associated with a breadth of cellular processes.

**Figure 1:**
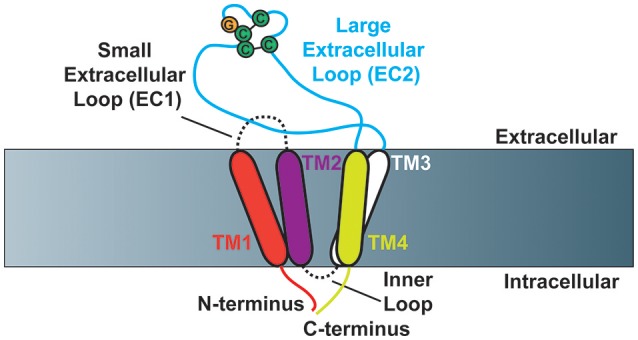
**Schematic of tetraspanin molecular structure (Based on Zimmerman et al., 2016)**. Cartoon depicting the structural characteristics of tetraspanins. Tetraspanins have four transmembrane domains (TM1-TM4), which create one small (EC1) and one large (EC2) extracellular loop as well as a short inner loop. The N- and C-termini of tetraspanins are localized to the intracellular side of the membrane. The Cys-Cys-Gly amino acid motif is depicted in addition to the two characteristic disulfide bonds that are formed in EC2.

Members of the tetraspanin family of proteins have four transmembrane domains, which contribute to the creation of a small (EC1) and large (EC2) extracellular loop (Figure 1). The large extracellular loop contains a conserved Cys-Cys-Gly amino acid motif (CCG-motif), as well as two other conserved cysteine residues. EC2 of CD81 was resolved using crystallography (Kitadokoro et al., 2001), where the authors demonstrated that the four conserved cysteine resides within EC2 promote the formation of disulfide bridges, as had been suggested by previous reports (Levy et al., 1991; Tomlinson et al., 1993; Maecker et al., 1997). Moreover, molecular modeling studies using the CD81 EC2 structure as a template predicted the topography of several other tetraspanins including CD37, CD53, CD82, and CD151 (Seigneuret et al., 2001; Seigneuret, 2006). These studies demonstrated that the EC2 domain of tetraspanins consist of one conserved and one variable domain, with the conserved domain consisting of a three-helix bundle while the variable domain is unique to particular tetraspanins. A recent report resolved a crystal structure of full-length CD81, finding that the four transmembrane domains create a cholesterol-binding pocket (Zimmerman et al., 2016). Furthermore, the authors performed molecular dynamics simulations that suggest CD81 can adopt an open or closed conformation depending on whether or not cholesterol is bound.

**Figure 2:**
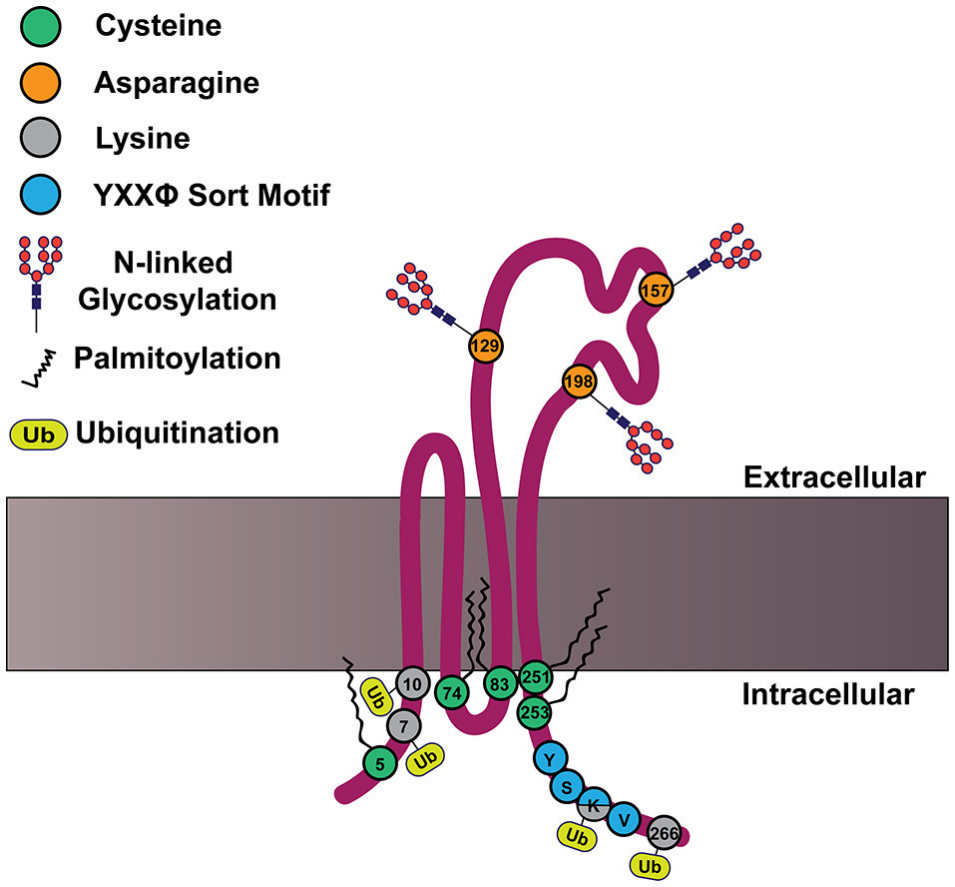
**CD82 structure and motifs**. Cartoon depicting CD82 topology within the plasma membrane and important motifs. CD82 contains five membrane proximal cysteine residues (shown in green) at residues 5, 74, 83, 251, and 253, which can be palmitoylated. There are three asparagine residues in EC2 (shown in orange) that are predicted to be N-linked glycosylated at residues 129, 157, 198. There are four cytoplasmic lysine residues 7, 10, 263, and 266 (shown in gray), which are predicted to be ubiquitinated. The C-terminal tyrosine based sort motif (YXXø) is depicted in blue at amino acids 261–264; for CD82 this motif is Tyr-Ser-Lys-Val.

In addition to the defining features of tetraspanins, many members of the tetraspanin family also contain post-translational modifications. For example, tetraspanins may be palmitoylated at membrane proximal cysteine residues, which was demonstrated to regulate protein-protein interactions (Berditchevski et al., 2002; Charrin et al., 2002; Yang et al., 2002, 2004). Meanwhile, tetraspanins can also be N-linked glycosylated at asparagine residues, which is less clearly understood (Ono et al., 1999; Stuck et al., 2012; Marjon et al., 2016). Tetraspanins may also be ubiquitinated at cytoplasmic sites, which contributes to their down-regulation (Lineberry et al., 2008; Wang Y. et al., 2012). An example structure of tetraspanin CD82 is depicted in Figure 2, with the post-translational modifications highlighted. How these tetraspanin post-translational modifications impact signal transduction will be addressed in more detail later in this review.

Through their function as molecular scaffolds, tetraspanins contribute to organismal development, reproduction, and immunity (Kaji et al., 2000, 2002; Le Naour et al., 2000; Miyado et al., 2000; García-Frigola et al., 2001; Levy and Shoham, 2005; Jarikji et al., 2009; van Spriel, 2011; Han et al., 2012). Consistent with their expression being primarily found in multicellular organisms, it is not surprising that many processes to which tetraspanins contribute center around cell-cell- interactions. Additionally, numerous tetraspanins are also associated with the development and progression of disease, in particular, with respect to cancer and cancer cell-niche interactions (Zoller, 2009; Hemler, 2013). Although tetraspanins do not have known adhesive ligands or catalytic activity, they contribute to cellular physiology by organizing molecules within the plasma membrane into microdomains.

The proposed function of tetraspanins is to organize the plasma membrane by facilitating the formation of what are termed tetraspanin enriched microdomains (TEMs). TEMs consist of homophilic and heterophilic interactions amongst tetraspanins, interactions between tetraspanins and other membrane proteins, as well as interactions between tetraspanins and proteins at the membrane/cytoplasm interface (Hemler, 2005; Charrin et al., 2009, 2014; Stipp, 2010). Moreover, these protein associations can occur through direct binding between tetraspanins and other proteins or through tetraspanin interactions with a common binding partner.

**Figure 3:**
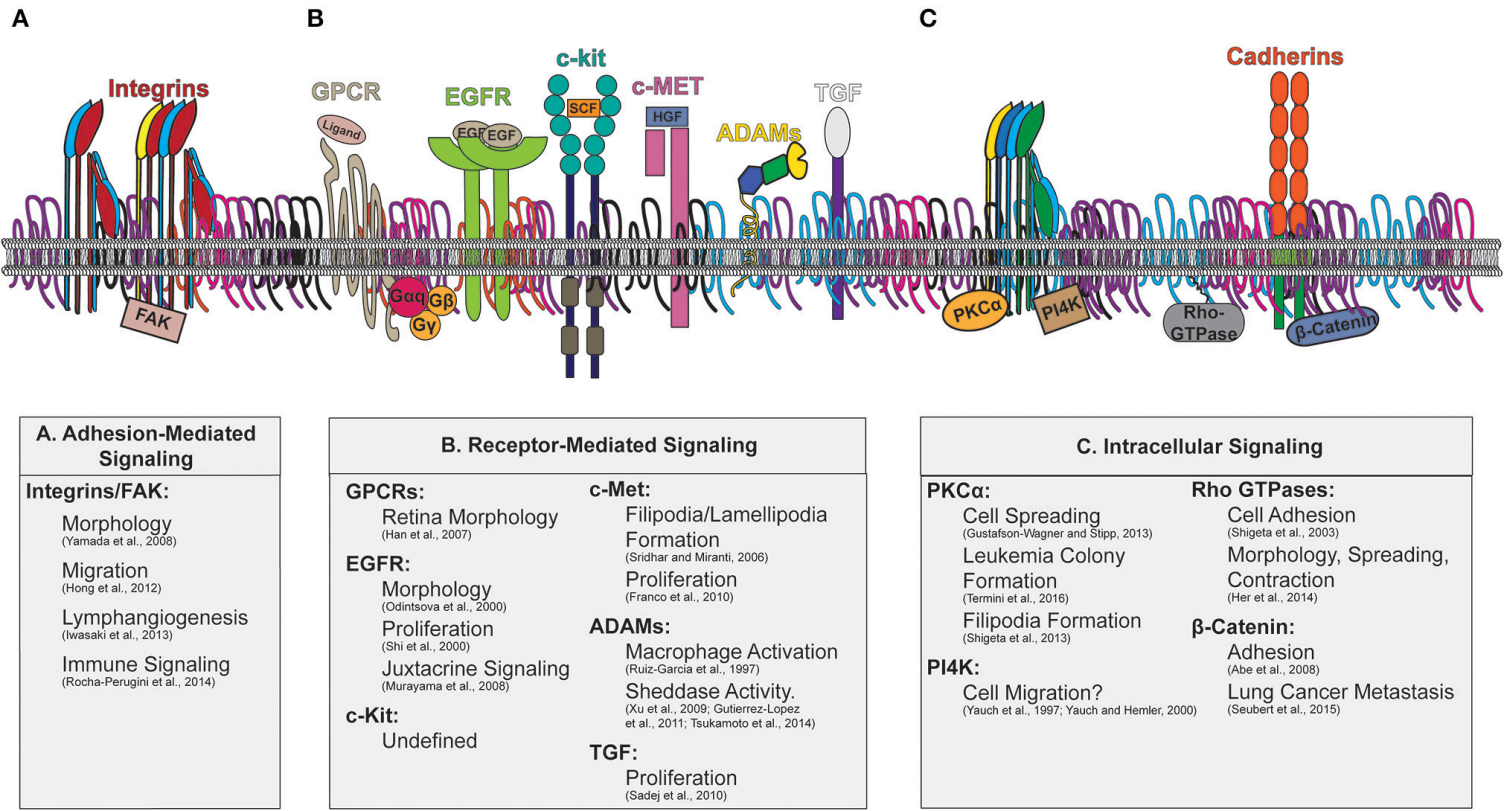
**Tetraspanin enriched microdomains with signaling molecules**. Illustration of the plasma membrane depicting tetraspanin interactions with membrane and cytosolic signaling molecules. The downstream signaling consequences attributed to tetraspanin regulation are indicated beneath. Key signaling molecules modulated by tetraspanins include: **(A)** Adhesion-Mediated Signaling (Integrins/FAK), **(B)** Receptor-Mediated Signaling (GPCRs, EGFR, c-kit, c-Met, ADAMs, TGF), and **(C)** Intracellular signaling (PKC, PI4K, Rho-GTPases, and β-catenin).

Interactions between tetraspanin and signaling molecules have been detected for various types of proteins, including adhesion and signaling receptors, and cytosolic signaling molecules, which are depicted in Figure 3. The downstream cellular consequences of these interactions vary, ranging from regulation of cellular adhesion, migration, contractility and morphology. As recent comprehensive reviews focused on tetraspanin regulation of immune signaling are available (Levy and Shoham, 2005; Halova and Draber, 2016), we will discuss other major classes of signaling molecules regulated by tetraspanins, as well as the cellular consequences of such regulations.

## Tetraspanins and adhesion-mediated signaling

One of the most prominent classes of adhesion receptors which tetraspanins are known to regulate is the integrin family of proteins. Integrins are heterodimeric proteins consisting of one α and one β subunit, and this combination of subunits dictates their ligand specificity (Humphries et al., 2006). Numerous studies identified direct and indirect interactions between integrins and tetraspanins (Slupsky et al., 1989; Rubinstein et al., 1994; Berditchevski et al., 1996; Mannion et al., 1996; Yáñez-Mo et al., 2001, 1998; Stipp and Hemler, 2000; Berditchevski, 2001). Though integrins lack intrinsic catalytic activity, they propagate signals through a variety of cytoplasmic signaling molecules, many of which are components of focal adhesions (Schwartz, 2001). Through a combination of imaging and biochemical studies, researchers showed that tetraspanins colocalize with the focal adhesion proteins vinculin and talin as well as myrstoylated alanine-rich C-kinase substrate (MARCKS), which is involved in PKC-mediated signaling (Berditchevski and Odintsova, 1999). Moreover, signaling downstream of integrins is also mediated by the focal adhesion kinase, which is further regulated by tetraspanins as indicated below.

### Focal adhesion kinase

Focal adhesion kinase (FAK) is a cytosolic protein which can interact directly with the integrin cytoplasmic tail, thereby allowing integrins to link to the actin cytoskeleton and promote downstream signaling (Schlaepfer et al., 1999). Immunoprecipitation studies demonstrated that tetraspanins CD9, CD63, CD81, CD82, and CD151 interact with the phosphorylated form of FAK (Berditchevski and Odintsova, 1999). Additionally, cells plated on anti-tetraspanin monoclonal antibodies demonstrated reduced FAK phosphorylation, further suggesting that tetraspanin scaffolding can contribute to FAK activation.

As suggested, a number of tetraspanins have been implicated in FAK regulation. It was shown that the siRNA knockdown of CD151 resulted in diminished phosphorylation of FAK, p130Cas, paxillin, and Src (Yamada et al., 2008). In fact, treatment with a CD151 monoclonal antibody, which reduced CD151 interactions with α3β1, also led to a reduction in FAK phosphorylation. In an attempt to rescue this phenotype, control or CD151 knockdown cells were treated with a β1 integrin activating antibody and these data demonstrated that FAK phosphorylation could not be rescued under enforced integrin activation. As such, this study provides evidence that tetraspanins may regulate integrin-mediated signaling through a mechanism independent of initial integrin activation. The authors quantified FAK autophosphorylation (Tyr397), which is a FAK modification stimulated by integrin clustering (Schlaepfer et al., 1999). As tetraspanins have been previously demonstrated to regulate integrin clustering (van Spriel et al., 2012; Termini et al., 2014), perhaps the loss of CD151 diminishes integrin clustering, thereby reducing FAK phosphorylation. Additionally, the presence of CD151 increased FAK and Src phosphorylation in response to plating on extracellular matrix components, which modulated GTPase activation and downstream cell migration (Hong et al., 2012). The authors demonstrated that there is a greater increase in FAK and Src activation in response to plating on laminin than fibronectin, which is consistent with previous findings that CD151 is closely associated with laminin binding integrins (Berditchevski et al., 2002; Stipp, 2010).

Another tetraspanin identified to regulate FAK activity is CD9. In the case of lymphatic dermal endothelial cells, CD9 knockdown diminished FAK phosphorylation in response to VEGF-1 administration, demonstrating that tetraspanin regulation of FAK signaling can occur through multiple activating stimuli (Iwasaki et al., 2013). The authors further demonstrated that this CD9-mediated reduction in post-adhesion signaling impaired lymphangiogenesis. Consistent with previous studies of CD151 (Yamada et al., 2008), Rocha-Perugini et al. demonstrated that silencing of CD151 or CD9 reduced the expression of phospho-FAK and phospho-ERK in response to T-cell engagement (Rocha-Perugini et al., 2014). A decrease in the accumulation of activated β1 integrins and phospho-FAK was also detected at the immune synapse in CD9 and CD151 knockdown cells, suggesting that CD9 and CD151 promote the recruitment to and retention of integrins at the immune synapse, which results in diminished integrin downstream signaling. Therefore, the influence that tetraspanins have on integrin localization provides a critical means to regulate integrin-mediated signaling.

Though not technically considered a tetraspanin, the L6 tetraspan protein, TM4SF5, has sequence characteristics and structural properties similar to tetraspanins (Wright et al., 2000). It was shown that the intracellular loop of tetraspan TM4SF5 is critical for promoting an interaction between TM4SF5 and FAK (Jung et al., 2012). The authors performed *in vitro* pull-down assays using the N- or C-terminal cytoplasmic regions of TM4SF5 or the TM4SF5 intracellular loop to assess FAK binding. It was found that only the intracellular loop interacted with FAK, although the precise sites of association remain unknown. Future studies focused on identifying the particular amino acid residues within tetraspans that promote this association may offer potential targets to attenuate FAK signaling, which can be deregulated in numerous types of cancer (Sulzmaier et al., 2014).

## Tetraspanins and receptor-mediated signaling

### G-protein coupled receptors

G-protein coupled receptors (GPCRs) are seven membrane-spanning proteins that transmit signals with the help of intracellular G proteins (Kobilka, 2007). Upon ligand binding, GPCRs can be coupled to Gα, Gβ, and Gγ subunits to activate numerous cellular responses including calcium and potassium channel regulation, as well as phospholipase C (PLC) and phosphoinositide 3-kinase (PI3K) signaling (Tuteja, 2009). With the use of model systems such as *Drosophila*, it was determined that tetraspanins can regulate GPCR-mediated signaling. For example, the *Drosophila*-specific tetraspanin, Sunglasses or Sun, is required for the light-induced down-regulation of rhodopsin, a light-sensitive GPCR (Xu et al., 2004). Interestingly, Sun was concentrated in the retina and removal of Sun resulted in retinal degeneration. Moreover, the authors determined that in flies with reduced Sun expression, extended exposure to light resulted in the diminished ability to down regulate rhodopsin. In line with these findings, Sun is most closely related to human tetraspanin, CD63, which is enriched within the lysosome (Metzelaar et al., 1991). Therefore, it is likely that Sun assists with GPCR signal attenuation by directing its endosomal trafficking in a similar manner to CD63. Additionally, an interaction between Sun and the Gq subunit of rhodopsin was identified, which was further proposed to help Sun promote the endocytosis of rhodopsin (Han et al., 2007).

The regulation of GPCRs by human tetraspanins has also been explored. It was shown that the GPCR, GPR56, associates with tetraspanins CD9 and CD81 (Little et al., 2004; Xu and Hynes, 2007), two tetraspanins which have also been demonstrated to interact with one another (Stipp et al., 2001). Through the use of mass spectrometry, it was also determined that the G protein subunits, Gα_11_ Gα_q_ and Gβ associate with CD81 and further immunoprecipitation studies demonstrated that this association is not detected with tetraspanins CD63 or CD151 (Little et al., 2004). The authors postulate that perhaps the regulatory role of tetraspanins with respect to GPCRs may be to enhance ligand binding and downstream signaling, though this has yet to be directly tested. Important future studies will involve the analysis of downstream signaling through tetraspanin-mediated changes in GPCRs, including the potential regulation of GPCR-ligand affinity.

### Epidermal growth factor receptor

In addition to GPCRs (Metzelaar et al., 1991; Xu et al., 2004; Han et al., 2007) and integrins (He et al., 2005; Winterwood et al., 2006; Termini et al., 2014), tetraspanins have also been demonstrated to regulate the trafficking and signaling downstream of the epidermal growth factor receptor (EGFR). EGFR is a transmembrane receptor that can be activated by numerous ligands including epidermal growth factor (EGF) and transforming growth factor-α (TGF-α). Ligand binding induces EGFR dimerization, which enhances EGFR catalytic activity (Jura et al., 2009; Valley et al., 2015). Moreover, EGFR endocytosis can serve as both a positive and negative regulatory signaling mechanism (Tomas et al., 2014). The contribution of tetraspanins in mediating EGFR trafficking has been extensively studied (Odintsova et al., 2000, 2003; Berditchevski and Odintsova, 2007).

Through a series of immunoprecipitation studies, it was shown that tetraspanin CD82 associates with EGFR and the overexpression of CD82 controls the phosphorylation kinetics of EGFR, Grb2, and Shc (Odintsova et al., 2000). It was determined that this regulation mediates the morphological response of HB2 cells to EGF stimulation. Interestingly, in cells expressing CD82, there was a more rapid down-regulation of EGFR upon EGF stimulation compared to cells that do not express CD82, indicating that CD82 contributes to EGFR down-regulation through modified internalization kinetics. This led the authors to suggest that the presence of CD82 modulates the signaling potency of the receptor even before it is activated. Furthermore, the authors speculate that the combination of reduced CD82 and increased EGFR expression may lead to uncontrolled signaling. Therefore, CD82, and likely other tetraspanins, may provide a means to attenuate signaling through modulations in EGFR trafficking. A follow-up study found that CD82 negatively regulates ligand-induced dimerization of EGFR, but does not affect the dimerization of ErbB2 or ErbB3 (Odintsova et al., 2003). Although the authors did not examine the downstream effects of altered dimerization, they suggest that the differential compartmentalization of EGFR by CD82 might alter cellular signaling.

Further studies examined the role of the vesicular associated membrane protein (VAMP), TI-VAMP, and CD82 in regulating the surface dynamics of EGFR. In this study, knockdown of CD82 led to increased EGFR endocytosis upon EGF stimulation through increased AP-2 recruitment (Danglot et al., 2010). Furthermore, CD82 knockdown also altered the EGFR diffusion patterns on the plasma membrane and reduced ERK phosphorylation upon EGF stimulation, providing evidence that tetraspanins can regulate the spatial dynamics of proteins for controlling downstream signaling. This report provides a unique mechanism by which CD82, through cooperation with TI-VAMP and AP-2, can regulate EGFR signaling and surface dynamics. Moreover, the authors propose that these regulatory mechanisms may be in part controlled by CD82-mediated alterations in actin dynamics or the membrane lipid composition.

EGFR regulation by CD82 was also shown to mediate ganglioside production. More specifically, the overexpression of CD82 in combination with inhibition of ganglioside production resulted in increased EGFR phosphorylation in response to EGF stimulation (Li Y. et al., 2013). The authors speculate that significant interplay occurs between glycosphingolipid enriched microdomains and TEMs, which cooperatively regulate cellular signaling. The overexpression of CD82 might promote EGFR clustering, which may stimulate dimerization and thereby enhance downstream EGFR signaling. Alternatively, the reduction in ganglioside production might improve EGFR phosphorylation by reorganizing the receptors into clusters within TEMs, since gangliosides have been demonstrated to contribute to TEM organization (Odintsova et al., 2006).

Beyond the prominent role of CD82 in regulating EGFR, additional studies also identified CD9 as a mediator of EGFR signaling. With the use of an autocrine system of MDCK cells co-expressing CD9 and TGF-α, TGF-α stimulation promoted EGFR activation (Shi et al., 2000). The authors also utilized a paracrine system whereby CHO cells expressing TGF-α alone or TGF-α and CD9 together were plated with 32D cells expressing EGFR. This experiment demonstrated that co-expression of TGF-α and CD9 increases EGFR activation, although the precise mechanism by which CD9 modulates EGFR signaling remains unclear. Regardless, this study provides unique insight into how CD9 may regulate cellular signaling initiated through contact between adjacent cells. Interestingly, another report investigated the effect of CD9 expression on EGFR signaling, finding that increased expression of CD9 resulted in decreased phosphorylation of EGFR, Shc, and total Grb2 expression (Murayama et al., 2008). Though these studies demonstrate opposing effects of CD9 on EGFR, they also indicate that TNF-α plays a role in mediating EGFR activation through CD9. These studies open the possibility that other tetraspanins such as CD82 may also work in concert with TNF-α, similar to CD9 and TNF-α in mediating EGFR activation. Therefore, future analyses would benefit from examining the interplay of TNF-α with other tetraspanins in regulating EGFR signaling.

### c-Kit

The stem cell factor receptor or c-Kit (CD117) is a receptor tyrosine kinase that binds to its ligand, stem cell factor (SCF), which is also known as steel factor (SLF) or kit ligand (Lennartsson and Rönnstrand, 2012). c-Kit signaling can activate several signaling cascades, including PI3K, Src family kinases, and MAPK to name a few. Moreover, c-Kit mediated signaling can control numerous cellular processes including migration, survival and the differentiation of hematopoietic progenitor cells. With the use of immunoprecipitation studies, it was determined that c-Kit associates with tetraspanins CD9, CD63, and CD81 and this interaction was enhanced upon treatment with SCF (Anzai et al., 2002). Although the authors found increased phosphorylation of c-Kit within the immunoprecipitated fraction, they determined that this does not enhance kinase activity in response to SCF treatment. Rather, the kinetics of SCF binding to c-Kit were altered when c-Kit associated with CD63. The authors suggest that this might be because free c-Kit is internalized upon SCF binding, implying that perhaps the CD63/c-Kit complex is more stable on the cellular surface. While this study alludes to a role for tetraspanins in regulating c-Kit phosphorylation, further analysis is necessary to determine the downstream consequences of tetraspanin mediated c-Kit activation. Additionally, the possibility that tetraspanins, such as CD63, might stabilize c-Kit and modulate signaling through alterations in protein trafficking could have significant impact on specific leukemias where c-Kit expression and activation are known to be dysregulated (Ikeda et al., 1991; Goemans et al., 2005; Boissel et al., 2006; Corbacioglu et al., 2006; Paschka et al., 2006).

### c-Met

c-Met is a receptor tyrosine kinase that can activate numerous pathways to promote cellular survival, motility, and proliferation (Birchmeier et al., 2003). Hepatocyte growth factor (HGF) binding to c-Met causes c-Met dimerization, which helps to initiate various cellular signaling cascades including AKT, ERK/MAPK, and JNK (Organ and Tsao, 2011). Furthermore, the overexpression of CD82 diminished the phosphorylation of c-Met in response to integrin ligand engagement, resulting in reduced Src phosphorylation (Sridhar and Miranti, 2006). In the case of invasive tumor situations, the authors' data suggest that the loss of CD82 leads to enhanced activation of c-Met through integrin activation. Although the regulatory mechanism remains unknown, this study provides a clear indication that tetraspanins can modulate c-Met mediated signaling downstream of integrin engagement.

It was also shown through immunoprecipitation studies that CD82 and c-Met interact (Takahashi et al., 2007). Moreover, the authors demonstrated that upon the ectopic expression of CD82, activation of c-Met with HGF led to increased formation of lamellipodia and filipodia through modulations in GTPase activities. Additionally, the ectopic expression of CD82 also prevented c-Met association with Grb2 and PI3K, implicating that CD82 has an inhibitory role with respect to these binding events. As such, perhaps the Grb2 and PI3K binding sites within c-Met become inaccessible in the presence of the c-Met/CD82 interaction.

The regulatory role of CD82 with respect to c-Met-mediated signaling has also been extended to controlling ERK1/2 and AKT signaling in hepatocellular carcinoma cells (Li Y. et al., 2013). An alternative report focused on CD151 with respect to Met signaling, showing that knockdown of CD151 led to diminished HGF-induced proliferation (Franco et al., 2010). The researchers determined that CD151 knockdown decreased tyrosine phosphorylation of the β4 integrin subunit, which decreased MAPK signaling through ERK in response to HGF. Therefore, this study suggests that the c-Met-CD151-β4 complex is critical for MAPK signaling. While the molecular link between tetraspanins and ERK or AKT downstream of c-Met remains an open question, this work implicates integrins as a possible connection.

### Transforming growth factor signaling

Transforming growth factor α (TGF-α) is synthesized as a transmembrane protein, which can become cleaved by metalloproteinases to release soluble TGF-α (Pandiella and Massagué, 1991). This cleavage is stimulated by endotoxins (Breshears et al., 2012; Liu Z. et al., 2013) and ROS (Boots et al., 2009) and is mediated primarily by ADAM17 (Peschon et al., 1998), but also by ADAM10 (Hinkle et al., 2003) and MeprinA (Bergin et al., 2008; Minder et al., 2012; Singh and Coffey, 2014). Moreover, TGF-α can interact with and activate EGFR on neighboring cells (Schlessinger and Ullrich, 1992; Thorne and Plowman, 1994; Moral et al., 2001). An association between CD9 and transmembrane TGF-α was identified and found to be dependent on the TGF-α ectodomain (Shi et al., 2000). The experimenters illustrated that the cleavage of TGF-α was inhibited by CD9, implicating a role for the association between CD9 and TGF-α as a means of protecting TGF-α from proteolytic cleavage. The authors suggested that the inhibition of TGF-α cleavage feeds into enhanced TGF-α induced EGFR activation, which can increase cellular proliferation. This study provides evidence that tetraspanins, such as CD9, can promote cellular signaling by stabilizing transmembrane proteins, thereby providing a potent activation stimulus to mediate juxtacrine signaling. Protein kinase C (PKC) and MAPK signaling can also regulate TGF-α cleavage (Baselga et al., 1996; Fan and Derynck, 1999). As tetraspanins can regulate PKC and MAPK signaling (Zhang et al., 2001; Termini et al., 2016), a closer examination into the interplay between these molecules in mediating TGF-α signaling may provide a more comprehensive view of the complex regulatory networks at play within TEMs.

A follow up study demonstrated that CD9 expression enhances TGF-α expression at the cell surface using MDCK cells (Imhof et al., 2008). Here, CD9 was shown to promote the trafficking of TGF-α from the Golgi to the cell surface by stabilizing the glycosylated and prodomain-removed forms of TGF-α. Furthermore, the authors demonstrated that the expression of TGF-α and CD9 alters actin organization and focal adhesion formation, supporting the notion that the combination of CD9 and TGF-α expression produces dramatically different signaling responses than the expression of TGF-α alone. Therefore, the tetraspanin expression profile should be considered when characterizing TGF-α signaling, particularly in many cancers where TGF-α expression is thought to support cancer progression (Kenny and Bissell, 2007).

Additionally, the contribution of tetraspanins to the regulation of the TGF isoform TGF-β1 has been assessed. Researchers used CD151 knockdown MDA-MB-231 cells and determined that in the presence of TGF-β1, CD151 knockdown cells had a significantly decreased proliferative rate compared to control cells (Sadej et al., 2010). More specifically, in the CD151 knockdown cells, TGF-β1 stimulation led to reduced p38 phosphorylation, resulting in decreased metastasis. Mechanistically, the authors propose that CD151 modulations of the plasma membrane may alter the distribution of TGF-β1 receptors and downstream signaling. Future studies may focus on determining how CD151 modulates the molecular organization of the TGF receptor, as this may provide a mechanism to regulate downstream signaling.

### A disintegrin and metalloproteases

The A Disintegrin and Metalloprotease (ADAM) family of transmembrane and secreted proteins contribute to the regulation of cellular adhesion, migration, proliferation, and signaling (Seals and Courtneidge, 2003). As the name states, ADAMs contain a disintegrin and a metalloprotease domain. While the metalloprotease domain can cleave extracellular matrix (ECM) components and mediate ectodomain shedding of cytokines, growth factors, the disintegrin domain can interact with integrins. Recent comprehensive reviews provide insight on the role that tetraspanins play in regulating membrane proteases, with a particular emphasis on their role in regulating ADAM10 and ADAM17 (Yáñez-Mo et al., 2011; Matthews et al., 2016). Initial reports demonstrated that ADAM10 is associated with CD9, CD81, and CD82, indicating that ADAM10 likely exists within TEMs. Interestingly, treatment with anti-tetraspanin antibodies stimulated the release of TNF-α and EGF in an ADAM10-mediated manner. Furthermore, through mass spectrometry studies and extensive immunoprecipitation studies, Tspan12 was found to associate with ADAM10, which contributed to the ability of ADAM10 to process amyloid precursor protein for shedding (Xu et al., 2009). Using several mutated TSPAN12 constructs, this association was determined to be regulated by EC1, the C-terminal tail and TSPAN12 palmitoylation. More recent co-immunoprecipitation studies revealed that the subgroup of TspanC8 tetraspanins (Tspan5, 10, 14, 15, 17, and 33) interact with ADAM10 (Dornier et al., 2012). Additionally, ADAM17 was also found to associate with tetraspanin CD9 in leukocytes and endothelial cells, which diminishes ADAM17-mediated TNF-α and ICAM-1 shedding. Interestingly, CD9 can regulate the catalytic activity of ADAM17 with regards to shedding of LR11 in monocytes, promonocytes and B-lymphoblastoid cell lines (Tsukamoto et al., 2014). As ADAMs are implicated in regulating various cancer cell types (Mochizuki and Okada, 2007), the role of tetraspanins in regulating ADAMs in malignant cells will provide significant insight and perhaps a means to attenuate aberrant ADAM activity.

ADAMs are produced as immature, inactive, preforms in the endoplasmic reticulum. During trafficking from the ER to the plasma membrane, the enzyme's prodomain is removed and ADAMs are then rendered catalytically active (Seals and Courtneidge, 2003). Interestingly, it was determined that TspanC8 contributes to ADAM10 maturation and ultimately the stabilization of ADAM10 at the cell surface (Prox et al., 2012). Furthermore, Tspan33 knockdown in erythrocytes resulted in diminished ADAM10 surface expression. Meanwhile, ADAM10 surface expression remained unchanged in platelets, demonstrating that tetraspanin regulation of ADAM10 is likely cell-type specific (Haining et al., 2012). Additionally, the role of Tspan33 in regulating ADAM10 for the control of macrophage activation was recently explored (Ruiz-García et al., 2016). Researchers utilized Tspan33 overexpressing Raw 264.7 cells and demonstrated that increased Tspan33 expression results in increased ADAM10 processing, consistent with the earlier aforementioned studies.

## Tetraspanins and intracellular signaling

Although tetraspanins are known to primarily affect the properties of other membrane proteins, they have also been shown to regulate cytoplasmic signaling molecules. Signaling proteins are often recruited to the cytoplasmic interface of the plasma membrane where they initiate signaling and TEMs can serve as a potential membrane recruitment site. Therefore, in the following section, we will review how tetraspanins control the localization, kinetics, and signaling properties of cytosolic proteins.

### Protein kinase C

The protein kinase C (PKC) family of intracellular signaling proteins consists of isoforms, which are further classified into conventional, novel or atypical isoforms (Newton, 1995). PKCs can phosphorylate several targets, including the myosin light chain II (Liu X. et al., 2013), PKD2 (Waldron et al., 2001; Navarro and Cantrell, 2014), Ras GEFs (Jun et al., 2013), and the β1 integrin tail (Stawowy et al., 2005), which collectively contribute to the regulation of cell proliferation, apoptosis, and adhesion amongst other cellular behaviors (Kang, 2014). The interaction between tetraspanins and PKC was originally demonstrated in K562 cells using an elaborate series of immunoprecipitation experiments (Zhang et al., 2001). The experimenters used phorbol 12-myristate 13-acetate (PMA), which mimics diacylglycerol (DAG) to activate PKC (Castagna et al., 1982). Under PMA stimulated conditions tetraspanins CD9, CD53, CD81, and CD82 individually interact with PKCα and not with PI3K. Additionally, they determined that CD81 and CD151 associate with PKCβII. Moreover, in a PKCα pull-down, β1, α3, and α6 integrins were detected in complex with PKC. Therefore, it was suggested that tetraspanins serve to link PKC to integrins. In order to assess the tetraspanin domains that control PKC associations, chimeric mapping was performed by replacing portions of CD9 with portions of the non-PKC associating tetraspanin, A15/Talla1. These findings demonstrated that PKC association with tetraspanins occurs outside of the short inner loop, the large outer loop, and transmembrane 3 or 4.

A recent report also demonstrated that tetraspanin CD151 regulates skin squamous cell carcinoma through STAT3 and PKCα signaling (Li Q. et al., 2013). Utilizing wild type or CD151 ablated A431 epidermoid carcinoma cells, it was shown that the loss of CD151 reduces STAT3 activation in response to 12-O-Tetradecanoylphorbol-13-acetate (TPA) stimulation, which is another known activator of PKCα. The authors found that PKCα only associates with α6β4 upon TPA stimulation when CD151 is present. Together, these data suggest that perhaps the role for CD151 is to recruit PKCα into close proximity with the α6β4 integrin, which ultimately aids in the phosphorylation of α6β4. As such, these data build upon previous implications that tetraspanins link PKC to integrins (Zhang et al., 2001), but also provide evidence that this scaffolding is important for epidermal proliferation and STAT3 activation.

Another interesting report investigated how CD9, CD81, and CD151 expression affects PKCα association with TEMs (Gustafson-Wagner and Stipp, 2013). It was demonstrated that CD9/CD81 knockdown diminishes the ability for the α3 integrin to associate with PKCα, which delays cell spreading on laminin and directed migration. In contrast, CD151 knockdown enhanced the association of PKCα with the α3 integrin, while promoting cell migration on collagen-I. The authors propose that CD9/81 may serve as linkers of PKC to the α3 integrin subunit, or there might be an indirectly associating molecule at play. Furthermore, the authors propose that perhaps upon CD151 depletion, there is increased association between PKC and α3 due to the loss of CD151, which makes CD9/81 available to fully associate with α3, thereby promoting PKC-integrin association. This study provides substantial evidence that the roles of tetraspanins CD9, CD81, and CD151 are unique in their regulation of PKCα-integrin interactions.

Further examination into the regulatory role of tetraspanins with respect to PKC-mediated signaling has uncovered many unique cellular responses. For example, treatment of A431 cells with Calphostin C to inhibit PKCα reduced filipodia extensions as well as E-cadherin puncta formation, demonstrating the involvement of actin in tetraspanin-regulated PKC signaling (Shigeta et al., 2003). The authors suggest that CD151 directly or indirectly associates with PKCα, which they propose may activate Cdc42 to promote filipodia formation.

A more recent report from our laboratory demonstrated that CD82 regulates PKCα signaling in acute myeloid leukemia (AML) (Termini et al., 2016). Using quantitative FRET imaging and KG1a AML cell lines that overexpress wild type CD82 or a palmitoylation deficient form of CD82 (Delandre et al., 2009), we found that upon PMA stimulation, PKCα was recruited to the plasma membrane where it associates with CD82. However, upon extended PMA stimulation, this PKCα/CD82 association is reduced in cells overexpressing the palmitoylation deficient form of CD82, demonstrating that the palmitoylation of CD82 regulates the stability of the PKCα interaction. We went on to use super-resolution imaging to examine how the scaffolding properties of CD82 regulate the macromolecular clustering of PKCα and found that upon disruption of the CD82 scaffold, there is a significant reduction in the size of PKCα clusters. Moreover, using CD82 knock-down cells, we found that while PKCα is still recruited to the membrane upon PMA stimulation, large-scale PKCα clusters are not detected. This change in PKCα clustering was then linked to alterations in downstream ERK1/2 signaling that influenced the aggressive phenotype of AML (Termini et al., 2016). Interestingly, the kinetics of PKCα oligomerization were recently quantified and modeled using HEK cells where they found that the intramolecular clustering of PKCα contributes to downstream phosphorylation (Bonny et al., 2016). Collectively, these studies illustrate that the modulation of signaling molecule clusters may serve as an important regulatory mechanism for stabilizing and/or attenuating signal transduction pathways. Moreover, our work implicates tetraspanins as critical mediators of cluster size and stability. Future super resolution imaging studies focused on identifying how the clustering of tetraspanins can modulate downstream signaling through PKC and other molecules such as Rac or Cdc42 would be valuable to help clarify how tetraspanins and PKCα mediate cytoskeleton-dependent cellular responses such as adhesion and migration.

An interesting link was also discovered between PKC and EGFR-mediated signaling that is enhanced by CD82. c-Cbl is an ubiquitin ligase recruited to EGFR where it assists with receptor down-regulation (Joazeiro et al., 1999). The authors found that PKC mediates c-Cbl phosphorylation upon EGF stimulation in CD82 expressing H2B cells (Odintsova et al., 2013). The phosphorylation of c-Cbl serves as a negative regulator of enzyme function (Ryan et al., 2006), which may be responsible for inhibiting EGFR downregulation. Therefore, without CD82 present, EGFR can be quickly downregulated as PKC is not present to regulate c-Cbl. Collectively, these studies provide substantial evidence that implicates tetraspanins as signaling scaffolds that promote the close proximity of PKC with integrins, EGFR and cytoplasmic proteins like c-Cbl.

### Phosphatidylinositol 4-kinase

Phosphatidylinositol 4-kinase (PI4K) catalyzes the conversion of phosphatidylinositol (PI) to phosphatidylinositol 4-phosphate (PI4P), which is an important intermediate for lipid-mediated signaling (Clayton et al., 2013). A series of biochemical experiments demonstrated that PI4K exists within α3 integrin and CD63 containing TEMs (Berditchevski et al., 1997). The authors suggest that perhaps TEMs are responsible for linking the α3β1 integrin to PI4K. A follow up study from the same group explored this further, demonstrating that immunoprecipitation of α3 or CD151 yields similar levels of PI4K activity based upon PI4P production (Yauch et al., 1997). Additionally, using cells with diminished α3 expression, CD151 was pulled down, demonstrating that there is still PI4K associated with the complex. Conversely, immunodepletion of CD151 resulted in significantly diminished lipid kinase activity associated with α3, while CD63 and/or CD81 deletion did not have as significant of an effect. Collectively, these data implicate CD151 as a critical linker between PI4K and α3β1, which the authors suggest may support cell migration.

A subsequent follow up study demonstrated that PI4K associates with tetraspanins A15/TALLA1, CD63, CD151, CD9, and CD81, however it does not appear to associate with NAG-2, CD53, CD37, or CD82 (Yauch and Hemler, 2000). Moreover, PI3K and PI4P5K activity were not detected in CD63, CD81, and CD151 complexes, indicating that perhaps the association is specific to PI4K. Studies with CD9/CD82 chimeras were unsuccessful at determining the site of association with PI4K. Therefore, a closer examination into the structural domains within tetraspanins that contribute to their association with PI4K could provide insight into the mechanism by which tetraspanins may regulate the catalytic activity of PI4K and downstream responses.

### GTPases

Rho GTPases mediate signal transduction by switching between a GTP-bound (active) and GDP-bound (inactive) state (Bishop and Hall, 2000). There are numerous effector proteins downstream of GTPases including PI3K, PI-4-P5K, MEKK1, and DAG kinase. The Rho family GTPases Rac1, RhoA, and Cdc42 as well as the Ras family of GTPases translocate to the plasma membrane upon activation (Collins, 2003), where their regulation by tetraspanins continues to be defined.

For example, CD151 was demonstrated to regulate Cdc42 for the control of cellular adhesion. Using A431 cells, CD151 antibody treatment or CD151 overexpression was found to increase Cdc42 activation, which the authors suggest controls actin reorganization, promoting filopodia-based adhesions (Shigeta et al., 2003). Another study assessed how the coexpression of CD9 and TGF-α regulates GTPase signaling, finding increased and decreased levels of activated Rac1 and RhoA respectively, with Cdc42 levels remaining unchanged upon coexpression of CD9 and TGF-α (Imhof et al., 2008). This shift in signaling was determined to be due to enhanced EGFR signaling, which ultimately contributed to enhanced stress fiber formation. Additionally, the overexpression of CD82 was shown to decrease the proportion of GTP-bound Rac1, while RhoA and Cdc42 levels remained unchanged (Liu et al., 2012).

Previous work also demonstrated that CD151 promotes the association between CD151-β1 complexes and Ras, Rac1 or Cdc42. Immunofluorescence imaging showed that CD151 regulates the translocation of Rac1 and Ras to the membrane and promoted colocalization with β1 integrins (Hong et al., 2012). Interestingly, through the use of a CD151 chimera with disrupted α3β1 integrin association, the authors showed that this mutant is unable to recruit Rac1 to the membrane. Therefore, integrins also have the capacity to link GTPases to tetraspanins in a manner similar to what was previously proposed for PKC and tetraspanins (Zhang et al., 2001; Li Q. et al., 2013). An association between Rac1 and the C-terminal, cytoplasmic region of CD81 has also been suggested based on the use of an eight amino acid C-terminal tail peptide (Tejera et al., 2013). Future experiments that mutate or delete the CD81 C-terminal tail will be important to demonstrate that such a mutation eliminates Rac1 association, further validating the interaction. Furthermore, upon EGF stimulation, it was shown that knockdown of CD81 increases Rac activation. A more recent study identified a correlation between CD9 expression and GTP bound Rac1 expression in acute lymphoblastic leukemia patient samples (Arnaud et al., 2015). Moreover, this group also determined that the C-terminal tail of CD9 is important for regulating Rac1 activation. Interestingly, the C-terminal region of CD9 has two known palmitoylation sites (Charrin et al., 2002), and Rac can also be palmitoylated (Tsai and Philips, 2012). Therefore, it is possible that these post-translational modifications may help to anchor tetraspanins and GTPases into similar membrane compartments.

Tetraspanin regulation of RhoA signaling, which can promote changes in cytoskeletal organization, has also been characterized (Sit and Manser, 2011). Using human aortic smooth muscle cells, CD9 knockdown decreased the expression of GTP-bound RhoA, leading to defects in cellular morphology, spreading and contraction (Herr et al., 2014). The authors suggest that integrins are involved in CD9-mediated alterations in RhoA activation by possibly stabilizing integrin-ECM interactions, augmenting RhoA activation. Interestingly, a recent report demonstrates that the loss of CD151 in breast cancer cells resulted in increased RhoA activation as quantified using FRET biosensors (Novitskaya et al., 2014). These data are contrary to Hong et al. (2012), who showed no change in Rho activation upon CD151 depletion. However, the change in FRET efficiency detected was <5%, which would likely be below the detection of the small GTPase protein pull-down assays used by Hong et al. Moreover, a separate report demonstrated that the knockdown of CD151 in human dermal microvascular endothelial cells resulted in an increase in RhoA-GTP and decreased Rac1-GTP (Zhang et al., 2011). Future studies focused on the mechanism by which tetraspanins can modulate GTPase activation will be important for determining how certain tetraspanins may be targeted to control specific GTPase activities in specialized cell types.

### β-catenin

β-catenin is a component of the Wnt signaling pathway that binds to the cytosolic portion of cadherins to initiate cellular signaling (Valenta et al., 2012). Through this complex formation, β-catenin promotes the internalization and recycling of E-cadherin, thereby destabilizing the complex and ultimately reducing cell-cell adhesion. Researchers determined that ectopic CD82 expression in h1299 cells relocalizes β-catenin to E-cadherin at the cell membrane, which stabilizes complex formation (Abe et al., 2008). Furthermore, they showed that ectopic CD82 expression increased cancer cell aggregation. To assess the downstream consequences of altered β-catenin localization, the authors stimulated cells with EGF or hepatocyte growth factor (HGF), demonstrating that ectopic expression of CD82 diminished β-catenin phosphorylation. While β-catenin phosphorylation is known to destabilize the E-cadherin complex, the mechanism for tetraspanin involvement remains to be clearly defined. Based on our previous work with N-cadherin (Marjon et al., 2016), we speculate that the CD82 scaffold might contribute to cadherin clustering, which may stabilize β-catenin membrane interactions, thereby protecting β-catenin from phosphorylation and down-regulation.

More recently, CD63 was shown to stabilize β-catenin signaling. In this study, shRNA knockdown of CD63 decreased β-catenin protein expression levels, which was suggested to occur through diminished levels of inactive GSK3β, leading to increased levels of phosphorylated β-catenin (Seubert et al., 2015). Furthermore, decreased levels of the β-catenin targets, MMP-2 and PAI-1, were detected, demonstrating CD63-mediated changes in downstream β-catenin signaling. The authors went on to find that the reduced expression of CD63 diminishes the metastatic potential of lung cancer cells, while the overexpression promoted tumor aggressiveness. However, modulations in signaling induced by CD63 overexpression were not explored. A previous study provided evidence that disrupting the interaction between the α3β1 integrin and CD151 enhanced β-catenin phosphorylation (Chattopadhyay et al., 2003). Therefore, it is plausible that the combination of integrins and tetraspanins serves to stabilize β-catenin within TEMs.

## Tetraspanin post-translational modifications and signaling

### Palmitoylation

S-palmitoylation is the addition of a 16-carbon fatty acid chain, palmitate, to cysteine residues of either cytoplasmic or membrane proteins (Blaskovic et al., 2013). Palmitoylation of cytoplasmic proteins promotes membrane anchoring, while palmitoylation of membrane proteins facilitates trafficking and membrane organization. Palmitoylation has been confirmed for tetraspanins CD9, CD151 (Yang et al., 2002), CD81 (Delandre et al., 2009), and CD82 (Mazurov et al., 2007), however other tetraspanins also contain conserved cysteine residues that are predicted to be palmitoylated. The defined role for palmitoylation is to modulate TEM formation (Yang et al., 2004). Therefore, we took a closer examination of how tetraspanin palmitoylation contributes to the signaling that occurs downstream of TEM associated proteins.

For example, the expression of the palmitoylation deficient form of CD151 weakened its association with integrins (Berditchevski et al., 2002), resulting in diminished phosphorylation of AKT in response laminin-5 engagement. These data indicate that palmitoylation-mediated disruption of TEMs can reduce downstream signaling responses. Additionally, a palmitoylation deficient form of Tetraspanin12 was shown to have diminished association with ADAM10, resulting in decreased ADAM10 activity as assessed by APP shedding (Xu et al., 2009). Recent work from our lab has shown that overexpression of a palmitoylation-deficient form of CD82 diminishes PKC membrane stabilization, reducing ERK1/2 activation, and downstream leukemia colony formation (Termini et al., 2016). Collectively, these studies demonstrate that tetraspanin palmitoylation contributes significantly to the regulation of downstream cellular signaling. Intracellular signaling molecules such as Ras (Eisenberg et al., 2013), Rac (Tsai and Philips, 2012), and PKC (Ford et al., 1998) can themselves be palmitoylated to assist with their membrane anchorage. As tetraspanin palmitoylation is thought to regulate lateral protein associations within TEMs, perhaps tetraspanin palmitoylation functions in concert with the palmitoylation of cytoplasmic proteins to produce stable membrane interactions critical for sustained signaling.

### Glycosylation

Although the large extracellular loop of many tetraspanins has been demonstrated to have one or more potential N-linked glycosylation sites, little is known about the functional consequences of this post-translational modification. The N-glycosylation pattern of CD82 was recently identified using proteomics and glycomics, determining that there are three putative N-glycosylation sites (Wang H. et al., 2012). Previously, these sites were suggested to regulate apoptosis, however the researchers did not examine the signaling that led to these apoptotic changes (Ono et al., 1999). Interestingly, the photoreceptor-specific tetraspanin retinal degeneration slow (RDS) can also be glycosylated (Kedzierski et al., 1999; Conley et al., 2012). More recently, the function of RDS glycosylation was re-examined by expressing a glycosylation deficient version of RDS in mice, which identified differential functional outcomes in cones vs. rod photoreceptor cells (Stuck et al., 2015). Moreover, the authors determined that glycosylation regulates the formation of RDS complexes with another tetraspanin ROM-1, demonstrating that glycosylation can modulate tetraspanin complex formation. A recent report from our laboratory examined the role of CD82 glycosylation with respect to acute myeloid leukemia homing (Marjon et al., 2016). In this study, we demonstrated that mutation of the three glycosylation sites within CD82 to inhibit glycosylation resulted in increased AML cell homing to the bone marrow, which we linked to increased molecular packing of N-cadherin via super resolution imaging. Although we have yet to examine signaling deficits in cells with disrupted CD82 glycoslation, it is possible that these changes in the molecular organization of N-cadherin may modulate the activation or stability of p120 catenin or β-catenin signaling downstream of N-cadherin.

### Ubiquitination

Protein ubiquitination is important for regulating cellular signaling by selectively targeting proteins for degradation. Both CD81 and CD151 were shown to interact with gene related to anergy in lymphocytes (GRAIL), which promotes tetraspanin ubiquitination, ultimately downregulating surface tetraspanin expression (Lineberry et al., 2008). Interestingly, it was determined that these tetraspanins can only be ubiquitinated at their N-terminus. Through mutational studies, it was shown that mutation of K8 and K11 diminished the ubiquitination of CD81, while mutation of K8, K11, and K17 ablated the ubiquitination of CD151. More recently it was demonstrated that TSPAN6 interacts with the adaptor mitochondrial antiviral signaling (MAVs) in 293T cells to inhibit RIG-I-like receptor (RLR) mediated signaling (Wang Y. et al., 2012). The authors went on to show that induction of RLR signaling promoted the ubiquitination of TSPAN6 at K11, K16, and K43, which are sites found within the TM1 of TSPAN6. Additionally, the authors determined that TSPAN6 ubiquitination serves to inhibit the formation of the signalosome, effectively down-regulating RLR signaling. As ubiquitination can target proteins for degradation, we suspect that tetraspanin ubiquitination will be a regulatory mechanism to allow for specific and efficient attenuation of tetraspanin-mediated signaling.

## Concluding remarks

Tetraspanins and their formation into TEMs enable the compartmentalization of membrane receptors within the plasma membrane. In this review, we focus on how tetraspanins also serve to connect these membrane-associated molecules with intracellular signaling complexes. It is now clear that tetraspanins regulate diverse cell signaling pathways that impact a breadth of biological processes. However, though numerous signaling molecules have been demonstrated to associate with tetraspanins, the mechanisms by which tetraspanins precisely modulate signal transduction remains relatively undefined. Future studies focused on how domains and motifs within tetraspanins promote or perhaps attenuate cellular signaling will help us understand the specific mechanisms used by this family of proteins to control signaling. Many laboratories are now using sophisticated imaging techniques to provide novel insight into the spatiotemporal interactions mediated by tetraspanins and TEMs. These studies will help to define how the scaffolding properties of tetraspanins contribute to the formation, stabilization and dynamics of signal transduction complexes at the plasma membrane. Moreover, these studies may provide the needed insight to establish tetraspanins and TEMs as potential therapeutic targets for the modulation of aberrant signal transduction that mediates processes such as inflammation, wound healing, and various types of cancer.

## Author contributions

CT and JG wrote and edited the manuscript. CT created the figures. All authors reviewed the manuscript.

## Funding

This work was supported by an F31 Fellowship from the National Heart, Lung, and Blood Institute (F31HL124977 to CT), an American Heart Association Scientist Development Grant (13SDG14630080 to JG), and an NIH investigator grant (RO1HL122483-01 to JG). Support was also provided from NIH P50 GM085273 (B. Wilson).

### Conflict of interest statement

The authors declare that the research was conducted in the absence of any commercial or financial relationships that could be construed as a potential conflict of interest.
